# Exploring the Temporal Patterns of Dynamic Information Flow during Attention Network Test (ANT)

**DOI:** 10.3390/brainsci13020247

**Published:** 2023-01-31

**Authors:** Keyi Duan, Songyun Xie, Xin Zhang, Xinzhou Xie, Yujie Cui, Ruizhen Liu, Jian Xu

**Affiliations:** Northwestern Polytechnical University, Xi’an 710072, China

**Keywords:** EEG, ERP, network construction, dynamic information flow, attention network test (ANT)

## Abstract

The attentional processes are conceptualized as a system of anatomical brain areas involving three specialized networks of alerting, orienting and executive control, each of which has been proven to have a relation with specified time-frequency oscillations through electrophysiological techniques. Nevertheless, at present, it is still unclear how the idea of these three independent attention networks is reflected in the specific short-time topology propagation of the brain, assembled with complexity and precision. In this study, we investigated the temporal patterns of dynamic information flow in each attention network via electroencephalograph (EEG)-based analysis. A modified version of the attention network test (ANT) with an EEG recording was adopted to probe the dynamic topology propagation in the three attention networks. First, the event-related potentials (ERP) analysis was used to extract sub-stage networks corresponding to the role of each attention network. Then, the dynamic network model of each attention network was constructed by post hoc test between conditions followed by the short-time-windows fitting model and brain network construction. We found that the alerting involved long-range interaction among the prefrontal cortex and posterior cortex of brain. The orienting elicited more sparse information flow after the target onset in the frequency band 1–30 Hz, and the executive control contained complex top-down control originating from the frontal cortex of the brain. Moreover, the switch of the activated regions in the associated time courses was elicited in attention networks contributing to diverse processing stages, which further extends our knowledge of the mechanism of attention networks.

## 1. Introduction

Attention refers to the prioritized processing of information in the face of other information competing for limited cognitive resources, handling incoming stimuli, making decisions, and producing outputs [1,2]. Generally, the attentional processes can be conceptualized as a system of anatomical areas composed of three specialized networks [3,4], i.e., the alerting network, orienting network, and executive control network, which can be induced and investigated by the attention network test (ANT) paradigm [5,6].

Shreds of evidence from neuroimaging techniques, such as functional magnetic resonance imaging (fMRI), have provided anatomical neuronal activations of brain regions related to sensory events and response tendencies control of the three networks [3,7]. The fronto-parietal and insular-opercular network have been discussed regarding their role in alerting [8]. The source of the orienting effect has been traced in the ventral network of the parietal, frontal, and subcortical areas anatomically, including the temporoparietal junction (TPJ), identified as part of a network responsive to sensory events [9]. The role of the anterior cingulate cortex (ACC) has been debated in the executive control network [10]. Nevertheless, due to the second-level time delay of fMRI, the dynamic information exchange of neurons among brain cortices in the time domain cannot be captured precisely, restricting its power to probe the underlying mechanism of the neural substrate corresponding to each attention network.

Interestingly, various studies have proven that each of the three attention networks was related to specified neuronal oscillations by means of electrophysiological techniques, especially by electroencephalogram (EEG). Evidence from event-related potentials (ERP) studies indicated that the contingent negative variation (CNV) generated by response anticipation of cue preparation was correlated with a shorter reaction time (RT) [11], as well as the variation of EEG power spectrum in specific regions of brain [12]. There were findings of enhanced negativity by the alerting and orienting of visual attention around 180 ms [13,14], suggesting the attention-related neuronal response in the extrastriate cortex [15]. The variation of P300 potential was found to differ within 300 and 600 ms post-target stimulus between varied attention conditions, with enhanced frontal distributed activities [16] and reduced amplitude at the parietal [13] in relation to response conflict and inhibition [6]. Furthermore, executive control was found to exhibit more complex response-locked time-frequency patterns with the shifting of frequency components centered at the average reaction time (RT) of 500 ms, to process conflict [12]. To summarize, all the above results suggest that the functional information interaction between specific brain regions and their temporal patterns in attention networks may reflect the processing of specific attention mechanisms, and short-time neuronal information exchanges among the specific anatomical cortex of attention networks is essential to investigate.

Previous research has also shown that brain network properties are tightly associated with individual abilities in attention networks [7]. As a matter of fact, complex cognitive activities requiring integrated information processing have been proven to benefit from the high global efficiency of information transferred across the brain [17]. EEG, characterized by the recording of neuronal group discharging with high time resolution, has shown superior performance in exploring the dynamic brain network in the cortex during varied cognitive processes, including motor imagery [18], decision-making [19], P300 experiment [20], behavior impairment such as mental fatigue [21] and Tourette’s Syndrome [22]. However, it is still unclear how the idea of these three attention networks, in each associated time course under the ANT paradigm, is reflected in the specific dynamic information flow during the short-time topology propagation of brain, assembled of complexity and precision.

Therefore, on the basis of complex neural substrates of the time-frequency activities underlying each attention network, we hypothesize that the dynamic brain network profiles employing EEG can be used to explore the dynamic information exchanges involved in attention networks. Based on this assumption, in this study, we aimed to explore the temporal patterns of brain dynamic information flow in each attention network by probing their temporal patterns via EEG analysis. Specifically, we first conducted ERP study using ANT on participants, while these ERP components were tested, to take part in the attention network. Then, we constructed the dynamic network model in short-time-windows to probe the specific information flow that each attention network component has during different stages in its entire timeline. Our experimental results demonstrated that each of the three attention networks distinctly exerted their roles in attention activities by means of specific patterns of information exchanges between different brain regions. This study investigated the complex dynamic information flow patterns during attention at a finer temporal scale, which may provide a more in-depth reference for understanding the neural information processing mechanism of the attention networks.

## 2. Materials and Methods

### 2.1. Participants

Twenty healthy adults (mean age: 23.1 years, seven females, one left-handed) were recruited from the Northwestern Polytechnical University participants in EEG recordings. This sample size was comparable to those in published reports [23,24,25]. The suitability of the sample size was confirmed by power analysis using G*power software [26]. A sample of 20 participants would be large enough to detect a typical effect size (*d* = 0.52) with a power of 80% at alpha level 0.05 in one-sample *t*-test to estimate the effect of attention networks. None of these participants had a history of substance abuse other than tobacco smoking, or a history of psychiatric axis I disorder according to DSM-IV, and had never received any psychopharmacological treatment or had a history of severe medical or neurological disorders. All subjects gave written informed consent before participating in this study. The study protocol was approved by the Ethics Committee of the Northwestern Polytechnical University, and the study was conducted in accordance with the Declaration of Helsinki and its amendments.

### 2.2. Experimental Procedures

**Figure 1:**
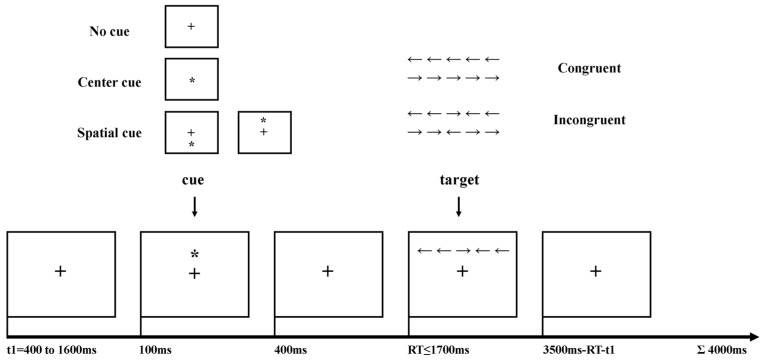
Schematic overview of the Attention Network Test (ANT) adopted in this study. The symbol * indicated the cue, and the symbol + represented the fixation in the center of the screen.

The experimental paradigm of the ANT was modified from Neuhaus [14], and the task design is displayed in Figure 1. The experiment was programmed in E-prime software. One fixation period (*t*1 = 400, 800, 1200, 1600 ms) was followed by an asterisk symbol warning cue occurring, including three cue conditions: no cue, center cue (at the fixation center of the screen), and spatial cue (above or below the fixation cross). Three types of cue conditions occurred proportionally with a duration of 100 ms. The target was an arrow flanked on both sides by two arrows either in the same direction (congruent condition) or in the opposite direction (incongruent condition), occurring with a maximum duration of 1700 ms after a 400 ms fixation period. Different trial types were pseudorandomized across three blocks of 96 trials so that each trial type had an equal probability of appearing before and after other specific trial. Subjects had to indicate the direction of the central arrow by left or right button press irrespective of flanker conditions. The RT of the subjects’ response was recorded in real time so that a variable fixation period (3500 ms–RT–t1) after target response assured the duration of each trial summing up to 4000 ms. Subjects were instructed to respond as quickly and accurately as possible during 96 trials of three blocks without any feedback. For better visual adaptability to the appearance of cue or target, the practice procedure was performed with a minimum of 24 trials along with feedback and EEG recording [14] with the help of the operator’s instruction [13]. The RT and response accuracy were recorded for each trial. Participants performed this task for almost 1 h or until they completed three blocks in total (288 trials). In addition, if the subjects were observed to have excess movements, the experiment would be stopped and the operator would instruct the subjects to relax for visual fatigue relief. Finally, due to a behavior label loss accident in one block, participants completed, on average 379 (98.70%) of the maximum 384 trials, ranging from 288 to 384.

### 2.3. EEG Data Acquisition and ERP Construction

Participants were seated individually in a quiet room with dim light. EEG recording was performed with a 64-channel Geodesic Sensor Net (Electrical Geodesics Inc, Eugene, USA.) with a 0.1–100 Hz bandpass filter and a sampling rate of 1000 samples per second digitized with a 12-bit A/D converter. During the entire experimental task, recordings in every channel were referenced to Cz with impedances below 50 kΩ. An additional scan of resting-state EEGs was recorded for 5 min before the entire experimental task.

**Figure 2:**
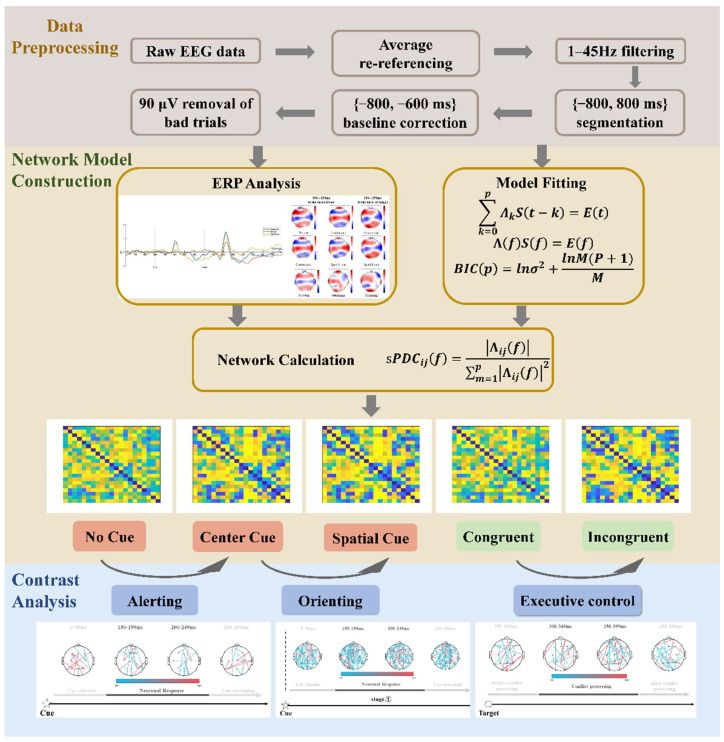
The pipeline of data processing, consists of EEG data preprocessing, ERP analysis, brain network construction and the contrast analysis for each of attention network.

Task-related EEGs were preprocessed with the following procedures as shown in Figure 2, including averaging referencing: 1–45 Hz bandpass filtering, [−800 ms, 1700 ms] (0 ms denotes target onset) data segmenting, [−200 ms, 0 ms] baseline correction, and ocular artifact trial removal (±90 μV as the threshold) [27,28], by the script modification from EEGLAB [29]. The mean number of artifact-free trials used for analysis was 69.90 (sd 16.00) for no cue condition, 70.85 (sd 14.43) for center cue condition, 70.80 (sd 15.02) for spatial cue condition, 104.65 (sd 23.28) for congruent target, and 106.90 (sd 23.53) for incongruent target.

Average referenced cue-related N1 (N1c), determined at POz, PO4, O1 and Oz [30], was defined as the first prominent negative peak within 150–200 ms after cue onset, while target-related N1 (N1t) was defined after target stimuli onset. The definition of cue-related P1 (P1c), also determined at the same electrodes [31], was the first prominent positive peak within 200–250 ms after cue onset, and target-related P1 (P1t) was after target stimuli onset. Target-related P300 was identified as a prominent positive deflection between 300 and 600 ms post-target stimulus and assessed at Cz, C2, C4, CP2, CPz, and CP4 channels to allow for analysis of executive control correlates [13]. The amplitude of these ERP components was defined as the mean amplitude in the 20 ms time window centered at the peak, and the corresponding latency was the time interval between the onset and the ERP peak.

The 2D current source densities (CSD) maps of each cue (no cue, center cue, spatial cue) and target condition (congruent, incongruent) were firstly computed in the averaged cue and target related-ERP components. Then, the differential CSD topographic maps of each attention network effects were calculated by the subtracted results of the averaged ERP components between the corresponding two conditions [13,14], for instance, CSD (alerting) = CSD (ERP _no cue_ − ERP _center cue_) within the time courses of N1c and P1c.

### 2.4. Fitting Model of EEG Signal

**Figure 3:**
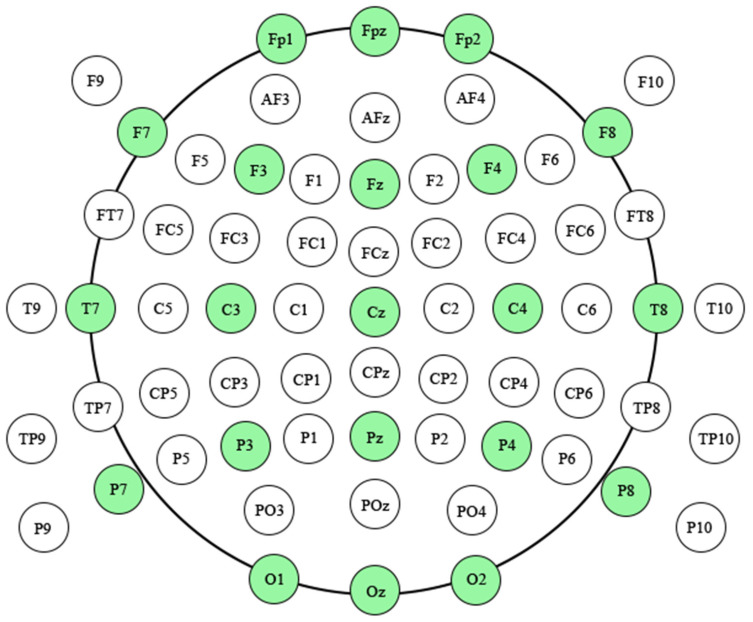
The horizontal distribution of scalp electrodes. The light green: canonical (21 out of 65) electrodes were used to construct the brain dynamic networks.

Considering the volume conduct effects, canonical (21 out of 65) electrodes in compliance with international 10–20 system were selected to construct the dynamic networks (FP1, FPZ, FP2, F7, F3, FZ, F4, F8, T7, C3, CZ, C4, T8, P7, P3, PZ, P4, P8, O1, OZ, O2), as shown in Figure 3.Considering of the Granger Causality theory [32] and the multi-channel of EEG signal, these artifact-free EEG signals were required to be fitted by mathematic model. In this study, these EEG time series in a short overlapping time window would be fitted by multivariate parametric model, in consideration of ERP treated as an ensemble of locally stationary segments [33] and the fact that multivariate measures outperformed bivariate measures especially in case of mutually interdependent channels [34].

Let $S\left(t\right)={\left[s_{1}\left(t\right),s_{2}\left(t\right)\ldots s_{N}\left(t\right)\right]}^{T}$ be a *N*-channel EEG time series in a short overlapping time window, while *t* represents the timing point. The process *S*(*t*) was stationary and can be described by the following multivariate autoregressive (MVAR) model:

(1) $$\sum_{k=0}^{p}\Lambda_{k}S\left(t-k\right)=E\left(t\right)$$

where $\Lambda_{1},\Lambda_{2},\ldots ,\Lambda_{p}$ were N×N coefficient matrices with $\Lambda_{0}=I$ and $E(t)={\left[e_{1}(t),\ldots ,e_{N}(t)\right]}^{T}$ was a zero mean uncorrelated noise vector with covariance matrix Σ. k represented the time delay, and the S(t−k) indicated the time series in the past k time. Once the *p*-order MVAR model was estimated, it became the basis of subsequent spectral analysis, and so the equation can be written in spectral domain as

(2) Λ(f)S(f)=E(f)

where $\Lambda (f)=\sum_{k=0}^{p}\Lambda_{k}e^{-j2\pi f\Delta tk}$,and Δt was the temporal interval between two samples. E(f) was the transformation of E(t), and S(f) was the power spectra of S(t).

The model order *p* was automatically determined by Bayesian information criterion (BIC) criterion within the range of 1–10 [35], which was defined as

(3) $$BIC\left(p\right)=\ln\sigma^{2}+\frac{\ln M\left(p+1\right)}{M}$$

where $\sigma^{2}$ was the function of model order *p*, as the fitting residual variance. *M* was the length of time series S(t). And the fittest model order *p* corresponded to the lowest value of BIC.

### 2.5. Dynamic Network Construction

In order to probe the dynamic neural exchanges, the 50 ms-length window was elected, which could simultaneously preserve correlation variability and maintain the smoothness of the estimated spectral quantities [33], specifically that the dynamic brain network was reflected on the specific time courses extraction based on the 50 ms-length time window. In this study, considering that the assumption of Granger causality may exist among specific areas [34], the squared partial directed coherence (sPDC) [36] within a 50 ms-length window among 1–45 Hz (same frequency band for ERPs analysis) in conditions for each subject was calculated by the support of the HERMES toolbox [37]:

(4) $$sPDC_{ij}\left(f\right)=\frac{{\left|\Lambda_{ij}\left(f\right)\right|}^{2}}{\sum_{m=1}^{p}{\left|\Lambda_{ij}\left(f\right)\right|}^{2}}$$

where $\Lambda_{ij}\left(f\right)$ was the MVAR model coefficient in frequency domain between *i*-th and *j*-th node, obtained by Equation (1).

These sPDC matrices indicated the power density relationship between the investigated node and can be interpreted as the fraction of power density of the *j*-th node that had an influence on *i*-th node. After that, these matrices within each time window were averaged across all trials for each type of condition, constituting to the subject-level 21 × 21 sPDC matrices in each condition.

### 2.6. The Sub-Stages of Attention Networks

Moreover, after all the 50 ms-level sPDC matrices were constructed, several sub-stages were extracted from the constructed networks based on the extracted ERPs, considering that N1, P1 and P300 components had been verified as reliable indicator of attention networks [11,30,38], and the aim to probe the specific information flow of each attention network. Specifically, the time courses in alerting and orienting after cue contained 3 sub-stages, including ‘cue stimulus’, ‘neuronal response’ and ‘cue sustaining’ [39,40]. The “cue stimulus” period was defined as the 0–100 ms after the cue appearing based on the cue occurring time in ANT paradigm; the time courses of “neuronal response” stage was selected based on the latencies and peaks of N1c and P1c in alerting and orienting, and the term “neuronal response” was chosen to elicit the regulation of brain response to the presented external stimuli [20]; and in the “cue sustaining” period, where a participant was required to wait for the occurring of target with the ability to conceive vigilance as sustaining the tonic arousal level that is necessary to react quickly to the cue stimuli [41,42]. As to executive control effect which occurred only after te target, the time courses of conflict-related P300 generation included three sub-stages of ‘before conflict processing’, ‘conflict processing’, and ‘after conflict processing’, which was in line with [19,20].

### 2.7. Statistical Analysis

Inspired by the initial subtraction definition of three attention networks in cognitive behavior analysis [5], i.e., RT(alerting) = RT(center cue) − RT(no cue), RT(orienting) = RT(spatial cue) − RT(center cue), RT(executive control) = RT(incongruent) − RT(congruent), as well as the relative independence of each type of cue or target condition [3,6], the brain network of each attention network in this study was defined by two paired-condition contrast via *t*-test statistics strategy.

For instance, the brain network of executive control network was constructed by the statistical differential matrices in congruent and incongruent target condition. First, subject-level 21 × 21 sPDC matrices in each 50 ms-length time window was constructed in congruent and incongruent condition respectively. Then, based on the target-related P300 waveform generation [38], the time course of the executive control network was extracted as mentioned above, in which the brain activity was isolated by the statistical differential matrices between incongruent and congruent condition via one-tailed *t*-test analysis at 99.5% significant level with FDR corrected. Thus, the dynamic brain network topology of the executive control network consisted of 21 × 21 matrices in three sub-stages, indicating the dynamic information flow of different brain cortex. In order to demonstrate the extent of the significant dynamic network, colored lines were adopted in all figures where the corresponding *p*-values were sorted from largest to smallest, i.e., the cyan represents relative lower significance while the magenta represents relative higher as shown in the color bar.

Moreover, in consideration that the small-world properties had been studied thoroughly [43], the degree analysis among those properties was selected due to its strength in evaluating the importance of the node in whole network [19,20], aiming at verifying the above post-target pattern changes of sparse information flow. The degrees of each channel in time windows were calculated by using the following equation [20]:

(5) $${\mathrm{degree}}_{i}\left(t\right)=\sum_{j}a_{ij}\left(t\right),i,j\in N$$

where N is the set of all nodes (channels) in the network, $a_{ij}\left(t\right)$ is the connection from node *i* to node *j* in time window *t*, and $a_{ij}\left(t\right)=1$ indicates the existence of corresponding connection, or else $a_{ij}\left(t\right)=0$.

## 3. Results

### 3.1. Behavior Performance

**Figure 4:**
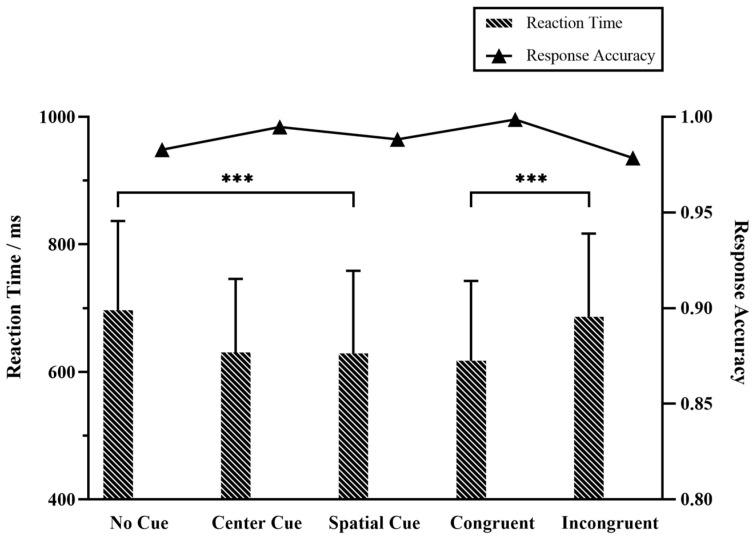
Behavioral main effects for reaction time and response accuracy during EEG recording. *** represented the significance *p* < 0.001 with repeated ANOVA test, and the error bars indicated the standard error of the mean.

The mean RT and response accuracy for each cue and target condition were shown in Figure 4, in which the mean RT was 651.56 ms (sd 132.35) and mean accuracy was 98.85% for all trials. We examined the effects of cuing types and flanker types on the reaction times when different cues were given. Repeated ANOVA of RT revealed significant main effects of different types of cues (F(2,57) = 23.18, *p* < 0.001) and flanker target (F(1,38) = 35.58, *p* < 0.001). Here, the post-hoc test indicated significant differences in the cue × flanker effect (F(5114) = 14.90, *p* < 0.001). On the whole, long RT was accompanied by relatively low response accuracy in different cue and target conditions. The no cue condition got the longest RT and lowest accuracy of the three cue conditions. The spatial cue achieved the fastest responses while unexpectedly the center cue led to the highest accuracy. The incongruent target impedes the response, i.e., longer RT and lower accuracy, in comparison with the congruent condition.

### 3.2. ERP Potentials Associated with Attention Networks

**Figure 5:**
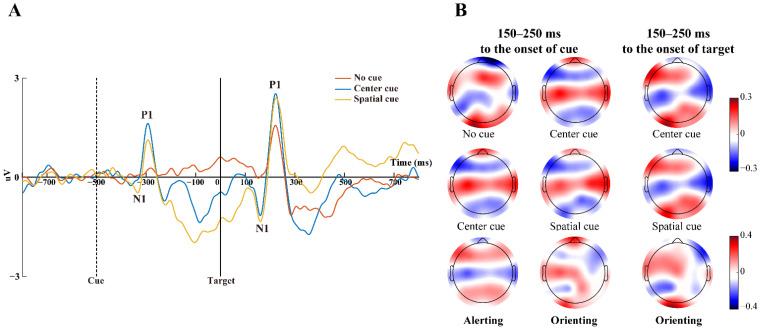
ERP waveforms and CSD topographical maps of each cue condition and two attention network effects: (**A**) grand average ERP waveforms stratified by cue and target conditions on electrodes POz, PO4, O1 and Oz. Cue stimulus onset is at −500 ms (dash line) and target stimulus onset is at 0 ms (solid line); (**B**) 2D top view of mean CSD maps of two attention network effects within 150–250 ms after cue and target onset. CSDs of alerting (bottom row) was obtained by subtracting averaged ERP of center cue condition (middle row) from no cue condition (top row), and orienting (bottom row) was obtained by subtracting averaged ERP of spatial cue (middle row) condition from center cue condition (top row). Units are $\mu V∕m^{2}.$

Alerting. The alerting was defined as the presence or absence of cues with no spatial information (center cue vs. no cue) [5]. As shown in Figure 5A, no cue condition did not induce any salient ERP potentials after the cue occurred, while the center cue evoked salient N1 and P1 potential (indicated as N1c and P1c), peaking within 200–250 ms to the cue onset. The N1c potential differentially impacted on parietal and occipital electrodes (POz, PO4, O1, and Oz), specifically post-hoc contrasts by paired *t*-test revealed significant differences of N1c latencies (*t*(19) = 2.187, *p* < 0.05), with significant difference of P1c amplitude and latencies (*t*(19) = 2.921, *p* < 0.05; *t*(19) = 4.783, *p* < 0.001). Parietal–occipital N1c and P1c latencies following the center cue were both significantly later, while the amplitude of the P1c was larger than the no cue condition.

Orienting. The orienting was examined by the presence or absence of cues with spatial information (spatial cue vs. center cue) [5]. And the related salient P1c peaked within 200–250 ms after the presentation of cues as shown in Figure 5A. Significant differences of N1c latencies (*t*(19) = 2.704, *p* < 0.05) were found for contrasts of orienting at parietal and occipital electrodes (POz, PO4, O1, and Oz). And as expected, parietal–occipital N1c latencies following the spatial cue was significantly later than the center cue condition. More interestingly, salient N1 and P1 potential appeared after the target onset (indicated as N1c and P1c), and so we further focused on the target-related potentials associated with the orienting network, however no significant differences were found in N1t or P1t at the parietal and occipital electrodes.

**Figure 6:**
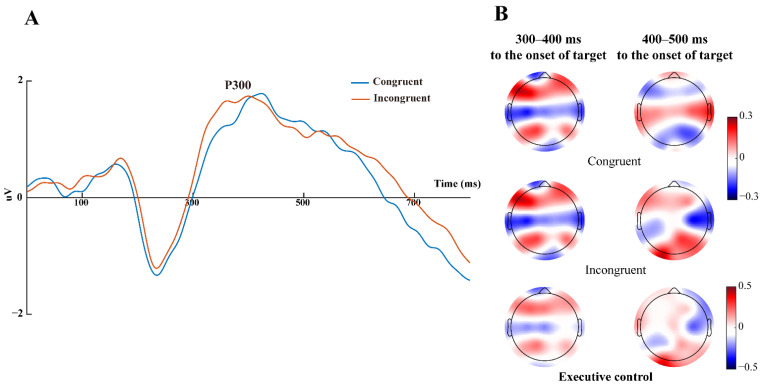
ERP waveforms and CSD topographical maps of 2 target conditions and executive control effect: (**A**) grand average ERP waveforms stratified by target conditions on electrodes Cz, C2, C4, CP2, CPz, CP4. Target stimulus onset is at 0 ms (solid line); (**B**) 2D top view of CSD maps of executive control effects within 300–500 ms after target onset (target-related P300). CSDs of executive control (bottom row) was obtained by subtracting averaged ERP of incongruent condition (middle row) from congruent condition (top row). Units are $\mu V∕m^{2}.$

Executive control. The executive control was defined by the effect between congruent and incongruent factors after the target onset [5]. The P300 component peaked within nearly 300–600 ms at Cz, C2, C4, CP2, CPz, CP4 shown in Figure 6A. However paired *t*-test indicated that there were no significant differences in P300 amplitude and latencies associated with executive control at these channels.

### 3.3. Dynamic Information Flow of Attention Networks

**Figure 7:**
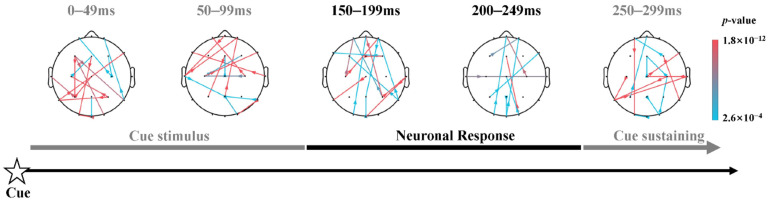
Dynamic information flow corresponding to the alerting network within 3 distinct sub-stages after the onset of cue. Stronger information flow induced by center cue condition compared to no cue condition (*t*(38) = 4.826, *p* < 0.001, FDR corrected).

Alerting. Based on the N1c and P1c waveform, the dynamic alerting network incorporated 3 after-cue sub-stages, i.e., center cue or no cue stimulus (0–99 ms), neuronal response (150–249 ms), and cue sustaining periods (250–500 ms), as shown in Figure 7, which indicated the dynamic topological connection patterns corresponding to the alerting network within three distinct sub-stages. To clearly illustrate the strength of significance of each connection in brain networks, the corresponding *p*-values were sorted from largest to smallest as shown in the color bar, i.e., the cyan represents relatively lower significance while the magenta represents relatively higher.

These networks exhibited sparse information flow after center cue occurred (*t*(38) = 4.826, *p* < 0.001, FDR corrected) compared with no cue condition. This kind of sparse characteristic was in accord with the small-world architecture of brain network where a combination of dense connected local hubs linked by more-sparse connections, with high modularity [44] and short path lengths [45]. As shown in Figure 7, the center cue evoked stronger interaction amid the anterior and posterior brain areas (*t*(38) = 4.826, *p* < 0.001, FDR corrected) right after its occurring, in comparison with no cue condition. And then during the neuronal response period, the alerting network exerted long-range interaction among prefrontal cortex and posterior cortex of the brain, thereafter converting to a regulating pattern of the occipital area. In addition, these networks elicited more compact patterns of information flow within 150–250 ms, which are in line with the N1c and P1c correlates. Then, in the cue sustaining stage, when subjects may be waiting for the occurrence of target nearly 250–299 ms after the onset of cue, there was sparsity of connections among the frontal cortex (FC), lateral occipital, and parietal involved in the alerting network.

Orienting. The dynamic topological network corresponding to the orienting network also included three sub-stages after the cue onset, i.e., center cue or spatial cue stimulus (0–99 ms), neuronal response (150–249 ms), and cue sustaining (250–500 ms). However, no sparse graph (*t*(38) = 2.998, *p* < 0.005, FDR corrected) with small-world property was found in the orienting network after the cue. These results were not conformed to the small-world property of brain network [46] and the definition of orienting [5,6]. Actually, previous evidences indicated an increase in gamma-band power (30–45 Hz) and some beta-band (14–30 Hz) components nearly 200 ms after spatial cue [12,47] onset, as well as an effect of gamma-band power increase for spatial cue minus center cue contrast in several dipole sources [12]. However, no sparse graphs within the frequency range from 1–45 Hz and only a low-gamma band (30–45 Hz) was found in the orienting network (*p* < 0.005, FDR corrected). Thus, considering that the gamma-band activity associated neural complexity may indicate a mixed-up situation that the target location may be exactly guided by the spatial cue [47] or the target tracking may require a shift of attention to the target location [12,48] (the spatial information validation in spatial cue condition), the 1–30 Hz frequency band was used to probe the post-target dynamic information flow of orienting network, to investigate the orienting effect thoroughly.

Thereafter, considering that the salient N1t and P1t correlated shows up, as shown in Figure 5A, we further focused on probing the dynamic information flow of the orienting network after the onset of target, and investigated the two sub-stages after the target onset, target stimulus (0–49 ms) and neuronal response (150–249 ms) [11,49]. As expected, the spatial cue did elicit a more sparse and salient flow of information exchange, served as post-target top-down and bottom-up networks (*t*(38) = 8.617, *p* < 0.005, FDR corrected), in contrast with the center cue condition.

**Figure 8:**
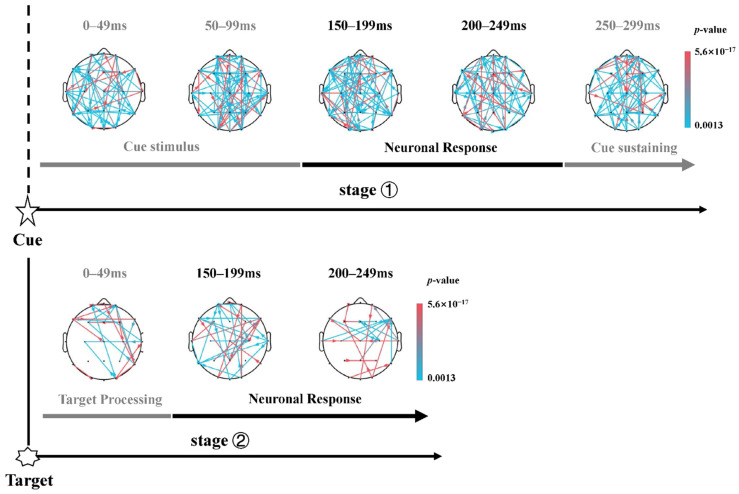
Dynamic information flow corresponding to the orienting network in six distinct sub-stages during two stages within frequency band of 1–30 Hz. Stronger information flow induced by spatial cue compared to center cue. Upper panel: dynamical brain network after the onset of cue (*t*(38) = 2.998, *p* < 0.005, FDR corrected). Lower panel: dynamical brain network after the onset of target (*t*(38) = 8.617, *p* < 0.005, FDR corrected).

Specifically, in the time courses after the target onset, the occurrence of a spatial cue induced stronger activation in the frontal cortex as sources, along with more activations of right brain regions, compared to the center cue. Then, during the early stage of neuronal response (150–199 ms), the pattern of information flow involved more regions of the cortex with more regulating sources, such as the right center brain area and the temporal cortex centered at the T8 channel. The switching of information flow pattern occurred at 200–249 ms (the lower panel of Figure 8), in which the right frontal cortex (rFC) is highly connected to other brain regions, along with occipital areas and frontal areas as weak information sources.

**Figure 9:**
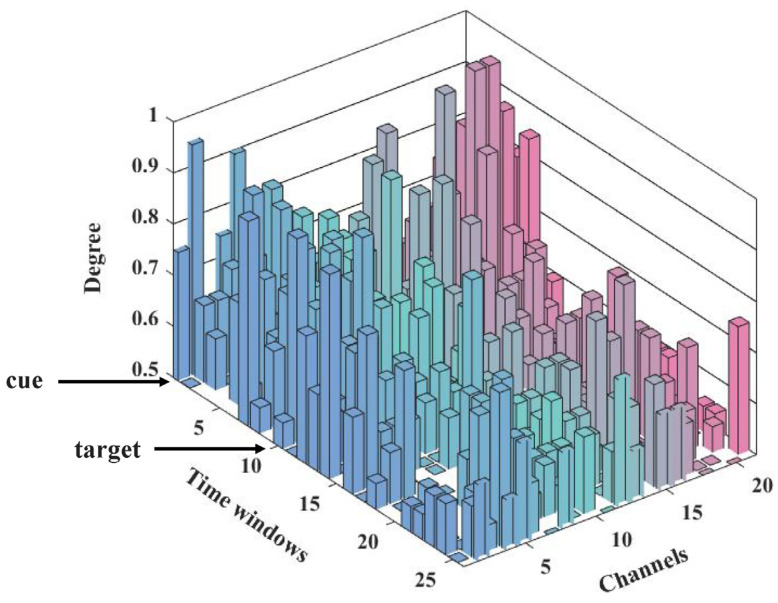
The degree of each channel during the whole-time courses of orienting. X axis: these channels numbers indicated the 21 canonical electrodes used in network construction, as shown in Figure 8. Y axis: the time windows with 50-ms length during 0–1300 ms to the cue onset. Z axis: the degree of orienting, each colored bar demonstrates the degree of specific network node (channel) in each time window, the values more than 0.5 were chose for comparing thoroughly.

In consideration of the above sparsity patterns of post-target information flow of the orienting network, the degree analysis of channels (network nodes) was performed to verify the pattern changes of information flows among post-cues and post-targets in orienting, due to its strength in evaluating the importance of the node in the whole network [19,20]. As shown in Figure 9, the degree of 21 channels was provided along with the time windows, corresponding to the whole-time courses of 0–1300 ms to the cue onset. To mark the time courses more concisely, the time windows were labeled by the corresponding sequence number of time windows with 50-ms length, e.g., the first number corresponded to the first time window (0–99 ms after the cue, i.e., the cue stimulus sub-stage in Figure 8); and the number 5 indicated the fourth time window during 200–249 ms. It should be noted that the onset of the cue and target corresponded to a scale of 1 to 10, respectively. The degree results in Figure 9 may demonstrate that, during the interval between cue and target, the information flows of the orienting network tend to play in a full-connected pattern, while after the target occurred, sparser network couplings emerged and confirmed.

Executive control. P300, occurring within nearly 300–600 ms after the target onset, is a reliable robust biomarker of executive control. Therefore, based on the target-related P300 waveform, the dynamic executive control networks involved three sub-stages after the target onset, i.e., before conflict processing (250–299 ms), conflict processing (300–399 ms), after conflict processing (450–499 ms). During the early processing stage (250–299 ms), the articulation nodes were distributed in the posterior cortex of brain, originating from the occipital areas, and spreading to related activation commands via the center area (CA). Afterwards, the frontal cortex served as a new source, regulating the response to the target stimuli during the whole conflict processing stage, especially indicating significant information flow originated from FC, along with activations within the parietal cortex (PC) and occipital cortex (OC) during 350–399 ms. The third stage was to act based on the preferences of the conflict processing stage, with left FC and CA activations, as well as a long-range connection between left FC and posterior areas.

## 4. Discussion

### 4.1. The Role of ERP Potentials Associated with Each Attention Network

The grand-averaged ERP potentials maps associated with each attention network were shown in Figure 5 and Figure 6. The alerting and orienting related cue or target-locked N1 and P1 potentials were, peaking at 150–200 ms and 200–250 ms, respectively, consistent with the previous study [50]. The modulation of the N1 potential was an early ERP component related to target stimuli [51], object discrimination [52] and recognition [53], occurring at a relatively early time point around 180 ms post-target onset [54] in the posterior cortex of brain [13], reflecting the way of offering a complex set of modulations both in alerting and orienting network [30]. Although not explored as extensively as N1, P1 was reported as part of the vertex potential or ‘vertex wave’ in conjunction with N1 [55]; however, exposing debates with N1 in a similar manner as that involved in attention demands [56]. Moreover, the target-locked P300 potential was related to the executive control network, occurring nearly between 300 and 600 ms before the target onset (Figure 6), in accordance with a previous study [13]. Referring to the P300 component occurred at frontal-central-parietal areas, its timing had been proven to be directly linked to response conflict [13] and response inhibition [57], originating from the stimulus-driven frontal attention mechanisms during task processing [58] and regulation to novel stimuli [6].

The above ERP results have elicited the neural correlates of each attention network to some degree, involving frontal, parietal, and the occipital regions which contributed to the topographical distribution of ERPs, in line with previous studies [12,30,38,47]. These interactions among different brain regions further prompt research in investigating the ERP-related neuronal courses in attention networks [59,60]. Thus, the neuronal stages associated with these ERP results of attention networks were worthy of deep investigation, such as probing the underlying specific diverse dynamic patterns of attention networks, especially the switch of the activated regions [7,22] in its time courses.

### 4.2. Temporal Patterns of Dynamic Information Flow in Alerting

The alerting network has been intensively studied, and there is substantial neuroimaging evidence about the ability of humans to maintain an alert and ready state [2] by affecting the rate at which attention can respond to stimulus [4,6]. The alerting network is fundamental for higher-order cognitive operations [61,62] and is dependent on efficient neuro-modulatory control [63]. The typical alerting contrast in the ANT used temporal cues to induce a state of alertness and thus taps primarily into the phasic component [3,5,7]. Thus, through a contrast statistical analysis (explained detailed in the Methods Part), the sparsity of dynamic information flow among brain regions in the alerting network, as shown in Figure 7, indicating the alerting effect was temporally exerted by center cue compared to no cue condition, possessing the characteristic of the small-world network [43,44,45].

During the earlier cue stimulus period, the center cue evoked significant long-range connections amid the anterior and posterior brain areas in comparison with the no cue condition after the cue occurred. This type of long-distance short-cut between spatially remote brain regions as high-cost brain network components [46,64], demonstrated the increase of information processing efficiency and benefits in aspects of adaptive human behavior [17,65]. Therefore, combining the related N1c and P1c potential (Figure 5) and the information flow during the earlier cue stimulus period (Figure 7), the center cue may instantaneously strengthens the information processing [63,66] through these long-distance interactions among brain regions, while no warning cue state may keep them remaining diffused across the two potential locations where the target would occur.

During the neuronal response period, the alerting network exerted tight information flow among frontal cortex and parietal cortex. Comparably and consistently, previous studies have shown that the lesions of the frontal and parietal lobe have effects on sustained attention, especially the decline of maintaining an alert state ability in the absence of a warning signal [67,68], thus the fronto-parietal pathways were verified with regard to their importance for alertness and spatial attention [68]. However, no salient right dominance was found in the dynamic information flow during each stage (shown in Figure 7), despite that some extensive imaging studies of both tonic and phasic alerting indicated a largely right hemisphere dominance of the brain in vigilance tasks [63,69].

In the time of nearly 250–299 ms after the onset of cue during cue sustaining stage, i.e., N1c and P1c component later, it seemed reasonable that subjects were waiting for the occurrence of the target [30]. Thus, FC was active, including frontal eye fields (FEF, BA 8) as the increased requirement of the task demand [70] and the anterior prefrontal cortex as the role of attention maintenance toward external stimuli [71]. This type of late modulation may be triggered by the neuronal activity induced by the center cue, which may be necessary for a generic motor preparation and sensorimotor integration by related CNV potential analysis [30] and fronto-parietal network activation in the same time-course [72]. Moreover, the involvement of the temporal and occipital regions was consistent with some neuroimaging studies [6,73], potentially as the basic cortical area in visual attention processing [74]. As the alerting network exerts effects immediately after the cue onset, the after-target time courses were not considered in alerting. Therefore, the sparse dynamic information flow during the cue sustaining stage may verify the hypothesis that the expectation about incoming stimuli was supposed to strengthen the correct prediction of sensory inputs [75] and the monitoring of significant events during task performance [30].

### 4.3. Sparser Information Flow of Orienting Network after the Target Onset

Most previous studies in orienting effects focused on the time courses post-cue/pre-target in ANT [11,76,77], corresponding to the initial orient definition of subtracted RT between the center cue and spatial cue [5,6]. Nevertheless, during the experiment, subjects were required to sustain the spatial cue until the target occurred where the two cue validity was contained. During this time, redundant information may be required for the delayed effect of neural complexity induced by spatial cue [48], and may explain the phenomenon that no sparse graph (*p* < 0.005, FDR corrected) was found in the after-cue orienting network, as shown in the upper panel of Figure 8 in spatial conditions. Especially when the target was miscued by the spatial cue, subjects had to break their focus of attention on the cued location and switch to the target location, identified by the interrupt signal allowing the switch to occur [3,78], associated with the involvement of TPJ and intraparietal sulcus (IPs) [79].

Thus, the underlying neuronal mechanism of this spatial cueing methodology may support that the probed post-target sparse dynamic information flow of the orienting network in our study (the lower panel of Figure 8), as well as its specific pattern changes of information flow via degree analysis (Figure 9), verifying its strength in evaluating the importance of the node in the dynamic information flow of orienting [19,20]. The post-target dynamic information flow of orienting included two sub-stages, in which the ‘target processing’ period corresponds to the short-term effect that the cue predicted later collection of a subsequent target [11] and the holding information online during later response anticipation [49], and the ‘neuronal response’ stage was based on the peaks and latencies of N1t and P1t components. Specifically, closely following after the target showed up, we found the activation correlated to the spatial cue was primarily in the FC as sources along with right hemisphere dominant (Figure 9). The prefrontal areas (frontal eye fields) [23,79], as hubs in the ventral attention networks and dorsal fronto-parietal network [7], have been proven to be involved in attention orienting. More involvement of right brain regions may explain the pathology mechanism of the neglect syndrome with strongly right lateralized lesions in TPJ, while the interaction between the TPJ and dorsal brain areas is also critical [80].

Later during the neuronal response stage, subjects were required to respond to the target after three types of cues occurring during the ANT paradigm, concentrating on target response preparation [11,12], confirmed by the dense connections among the cortex, as shown in Figure 8. After that, a switching network pattern of information flow from rFC to central and parietal regions indicated a top-down pattern [23,81] corresponding to the ERP generation, i.e., related information processing in visual cortices [51,82]. More interestingly, the occipital cortex involved in post-target time courses during subjects’ attention oriented to the selected cued items [83], may coincide with the fact that modulation of alpha power oscillation over occipitoparietal sites correlated to target perception and anticipatory visuospatial attention [84,85,86]. Thereafter, the specific situation subjects would encounter was the urgent need for executive control [11], i.e., solving the flanker conflict lying in the target. However, the interaction or activation in specific brain regions where spatial cue orienting would have an impact during the executive control is a complex neural correlate [77,87]. Previous studies have shown that, except for the frontoparietal network involved in the orienting network discussed previously [7], the cognitive-related cingulo-opercular network [9] as one of the two separable executive control networks proposed by Dosenbach et al. [82], was proven to exert function both in orienting and executive control network [2]. Moreover, the post-target sparse dynamic information flow of orienting network in 1–30 Hz frequency band indeed verified the complex situation of targets tracking where subjects encountered [12,47], due to the gamma-band oscillation induced by attended target [48] in the fusiform gyrus [7]. Together, these results and the present study suggest that the orienting network may exert its effect until the target occurred with intricate neural information exchanges.

### 4.4. Top-Down Processes of Response Conflict

**Figure 10:**
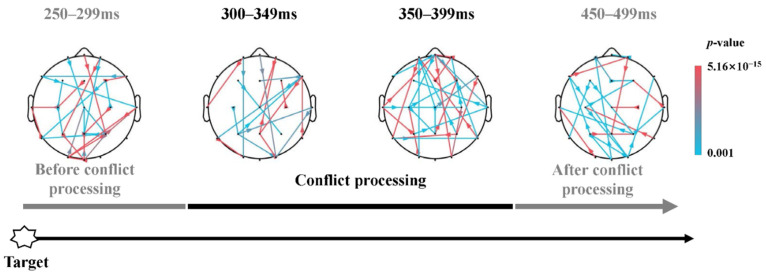
Dynamic information flow corresponding to the executive network within 3 distinct sub-stages after the onset of target. Stronger information flow induced by incongruent target compared to congruent target (*t*(38) = 3.585, *p* < 0.01, FDR corrected).

During the early stage before P300 occurred, subjects encountered the conflict situation of the target with flankers. As shown in Figure 10, the networks for the incongruent flankers during the before-conflict processing stage exhibited stronger couplings among various brain regions than the congruent, such as the occipital cortex and frontal regions [88], demonstrating the characteristics of information integration [89,90]. This integration to a large-scale complex extending across brain areas has been posited to be consciousness [91]. Therefore, we propose that nearly 250–300 ms post-target in the ANT paradigm, the exerting effect of the orienting network was followed by an information processing for the target conflict when subjects needed to perform conflict response and decision-making to a specific flanker type in incongruent trials rather than congruent trials.

Within the earlier stage of the conflict processing stage, FC was highly connected to other brain areas, along with PC activation and the lateral temporal cortex during 300–399 ms (Figure 10), to some extent, verifying the theory of a top-down control network [2,92]. Subsequently, there was a switch to a more impact pattern of information flow within 350–399 ms after the onset of target, with the prefrontal region as the information source linked up with most others in the cortex. This inspiring result was consistent with previous neuroimaging results that activation in ACC was relevant to executive control network [6,93], with a vital function in the case of target stimulus versus nontargets, conflict versus nonconflict, and errors versus correct [10]. However, how this complex pattern of top-down control exerted its effect still needs to be probed. The fronto-parietal activity may be interpreted as recruitment of cognitive conflict control [94], and error monitoring in decision-making [95]. This may suggest the top-down process control of visual attention and target-response conflict [96,97].

Thereafter, during 450–499 ms to the onset of target, i.e., pre-response activity (average RT 651.56 ms), activation in the frontal areas, parietal and occipital cortex was involved. Previous studies demonstrated that the posterior medial frontal cortex (MFC) is a hub within conflict resolution [98,99], and mid-frontal theta-band oscillations as the role of conflict monitoring and top-down control of sensory-motor decision-making by P300 analysis [60,100]. Moreover, the involvement of posterior parietal areas in conflict processing has been identified by their roles in spatial conflict tasks, and may be suggesting the conflict-processing engagement of mental adjustment [101].

## 5. Conclusions

In the current work, at a finer temporal scale, we probed the complex patterns of brain dynamic information flow in attention networks via ERP analysis and brain network model construction based on the EEG recording during the ANT paradigm. Our experimental results indicated explicitly that each of the three attention networks, i.e., alerting, orienting, and executive control, distinctly exerted their roles in attention activities by means of specific information exchanges between different brain regions during their time courses. In our future work, we will investigate closely the specific information flow during the period of spatial orienting effect, and how behavior changes will occur in human attention processing during neural regulation techniques such as electrical stimulation.

## Data Availability

Data supporting reported results can be required to corresponding author.
